# The ripple effect: impacts of climate change on menstrual health and paths to resilience

**DOI:** 10.3389/fgwh.2025.1569046

**Published:** 2025-04-24

**Authors:** Arundati Muralidharan, Marije Broekhuijsen, Lady Lisondra, Aeka Guru, Jacquelyn Haver, Sidra Irfan

**Affiliations:** ^1^ Global Menstrual Collective, London, United Kingdom; ^2^ Menstrual Health Action for Impact, New Delhi, India; ^3^ Programme Group Water Sanitation and Hygiene, UNICEF Headquarters, New York, NY, United States; ^4^ International Planned Parenthood Federation East, Southeast Asia and Oceania Region, Kuala Lumpur, Malaysia; ^5^ Yale University, New Haven, CT, United States; ^6^ School Health and Nutrition, Department of Education and Child Protection, Save the Children, Washington, DC, United States; ^7^ Menstrual Rights Global, Lahore, Pakistan

**Keywords:** menstrual health, menstrual hygiene, climate change, climate resilience, climate risks

## Abstract

Girls and women face greater threats and severe ramifications from climate change, with studies consistently finding that women experience more health risks from climate change than men do. Climate change endangers girls and women's sexual and reproductive health and rights, including their menstrual health and hygiene practices. However, menstrual health and hygiene is rarely discussed in the context of climate change. We scoped the existing evidence to describe the interlinkages between climate change and menstrual health and hygiene, and outline services that anticipate, respond to, cope with, recover from, adapt to or transform in response to climate related events, trends and disturbances. Specifically, we describe how climate change disrupts access to essential menstrual health and hygiene information, products and services, impacts menstrual disorders and the menstrual cycle, and intensifies existing inequalities. Recommendations for improvement include climate resilient menstrual health and hygiene services encompassing access to menstrual products and materials, impartment of knowledge and skills, access to climate resilient facilities and services, social support, and policy actions.

## 1 Introduction

Girls and women face greater threats and experience severe ramifications from climate change owing to its “multiplier” effect on existing forms of discrimination and vulnerabilities: poverty, socio-economic disparities, and inequitable gender norms (1–6). Studies consistently find that women face greater health risks from climate change than men do, undermining their sexual and reproductive health and rights (SRHR), and enhancing vulnerability to gender-based violence (GBV) (3, 7–11). As a part of SRHR, menstrual health and hygiene (MHH) requires attention as a vast majority of girls and women and people with gender diverse identities experience menstruation, and have associated needs that are directly and indirectly compromised by climate change events.

Menstrual health, “a state of complete physical, mental, and social well-being and not merely the absence of disease or infirmity, in relation to the menstrual cycle,” requires access to information and education, menstrual products, water, sanitation, hygiene (WASH) and disposal facilities and services, care for discomforts and disorders, positive social environments, and support to participate in all spheres of life without discrimination (12). While experienced by billions of people across the world as a healthy biological process, menstrual experiences are often accompanied by shame, stigma and discrimination, combined with limited knowledge and misinformation, and constrained access to essential menstrual products, WASH, disposal facilities and health services (13, 14). This negatively impacts health, girls' educational attainment, women's workforce participation, gender equality, and perceptions of self-efficacy (13, 15–17). Resultantly, MHH is increasingly seen as a health and human rights issue, and as intimately tied to SRHR, GBV and other gender equality issues given shared biological underpinnings, and socio-economic determinants across the life course (8, 12, 16, 18–22).

Emergency responses to environmental disasters and humanitarian crises seek to meet essential needs such as menstrual products and materials, supportive WASH infrastructure, and essential information (23–28). However, MHH is often under-prioritized in climate policies informing national and international priorities for adapting to and mitigating climate change. A recent review of 119 Nationally Determined Contributions (NDCs), the main vehicle for documenting climate commitments by nations signed onto the Paris Agreement, starkly revealed that about one-third incorporated attention to SRHR, and less than one percent integrated MHH (10). In comparison, climate resilient WASH services, a crucial requirement for MHH, are increasingly recognized across the enhanced NDCs, with 85% referring to water, and 45% to sanitation (29). Importantly, high income countries contribute more significantly to climate change, while low- and middle-income countries (LMIC) bear the brunt of climate change impacts (30). Therefore, with the climate crisis intensifying and affecting more people more frequently in LMICs, it is critical to understand how rapid and slow onset climate events affect MHH in daily life and in the long-term, and how relevant responses can be strengthened.

## 2 Methodology

Our paper seeks to:

• Outline the interlinkages between climate change and MHH, focusing on climate-related risks to MHH
• Describe MHH services that anticipate, respond to, cope with, recover from, adapt to or transform in response to climate related events, trends and disturbances

Through a desk review, we scoped the available evidence on interlinkages between climate related events, trends and disturbances, and MHH. This included documents on how rapid and slow onset climate change events affect menstrual needs and services, and how MHH interventions respond to climate related risks. The desk review was guided by the definition of menstrual health that outlines key requirements, and UNICEF's MHH program pillars (knowledge and skills; menstrual materials and supplies; facilities and services; and social support). It also builds on the normative definition of climate resilient WASH services (31) and the concept of climate-resilient development as described by the IPCC Sixth Assessment Report (AR6) (32). Our review also included journal articles and published reports on MHH during emergencies and humanitarian contexts given the links with climate change.

This paper uses the terms “girls and women” as the available literature focuses on these groups, but recognizes that persons with gender diverse identities also menstruate, and need to be considered while building understanding and responses. Further, the paper uses “menstrual health and hygiene” (MHH) to call attention to the holistic aspects of menstrual health, while acknowledging well-known actions and available evidence on menstrual hygiene management.

This paper was conceptualized and written by members of the Global Menstrual Collective's (GMC) Climate Change and Menstrual Health Working group. The GMC brings together multi-sectoral stakeholders working on menstrual health to foster collective, evidence-based advocacy to drive action and investment.

## 3 Findings

### 3.1 Climate change related risks to MHH

Climate change affects MHH through rapid onset, extreme or severe weather events (e.g., cyclones, heavy rainfall, heat waves, floods) that have immediate, adverse consequences for MHH, as well as through slow onset, long-acting climate events (e.g., rise in sea levels, rise in temperatures, desertification, salinization) that affect MHH directly and indirectly. We describe these risks.

#### 3.1.1 Climate change disrupts access to essential MHH information, products, WASH facilities and health services

Extreme weather events such as cyclones, floods, and wildfires damage homes, schools, health facilities, roads, WASH infrastructure and services, and cause power outages - all of which affect girls and women's ability to manage their menses (14, 33–36). Disrupted menstrual product supply chains and damaged distribution points (e.g., schools) leave people with limited or no safe products to use in the immediate aftermath of a disaster. Broken WASH facilities and affected water supply compromise hygienic management of periods (23, 25–27, 37). Hence, people may be forced to use available period products for a longer duration than is comfortable and hygienic, or resort to using unsafe materials. Where used, reusable cloth pads may not be washed thoroughly with clean water, dried properly, or stored safely. Regular genital cleansing or bathing can be challenging for many (27, 28). In the aftermath of disasters, girls and women may be forced to urinate, defecate and manage their menses in the open or in makeshift toilets with little privacy and safety. The challenges of changing, washing, drying, and discarding menstrual materials are worsened by prevalent social norms and taboos related to menstruation and menstrual blood as seen during the 2022 super floods in Pakistan (38).

Slow onset climate events also pose risks to MHH. Rising sea levels in coastal areas like Bangladesh lead to increasing salinity of freshwater sources, and more severe, recurrent and prolonged droughts in places like Somalia, Sudan, South Sudan, and the Sahel, limit water availability for various household activities and needs. Girls and women, often charged with household water collection, travel further to obtain clean water, and therefore prioritize water to meet household and other needs over their personal requirements.

Healthcare facilities and services are also damaged or overwhelmed with disaster response efforts, and affected by slow onset climate events, rendering them insufficient to address MHH needs (39–41).

#### 3.1.2 Climate change affects menstrual health related infections, disorders, and the menstrual cycle

Rapid and slow-onset climate events compromise menstrual health in ways that are still being understood. Most immediate and prominent are risks to menstrual hygiene practices, which lead to anxiety, physical discomfort, genital irritation, and reproductive tract infections (27). Climate events that cause temporary or long-term displacement increase girls and women's risk of GBV, the fear and/or experience of which curtails mobility and the ability to meet essential menstrual and other personal needs (7, 9, 42).

Slow-onset and chronic climate events like droughts, extreme heat, and saltwater intrusion affect the menstrual cycle, bleeding patterns, as well as urogenital discomforts and infections. Descriptive studies from climate vulnerable areas like Bangladesh find that girls and women bear the brunt of fresh water collection, reduce their water intake, and suffer genital irritation and infections when using menstrual cloth repeatedly washed in saline water (43, 44). Indigenous women who live in rural communities also undertake suboptimal menstrual hygiene practices due to limited water access in the dry season (45). Extreme heat can cause hormonal imbalances and intensify endocrine disorders and urinary tract infections (46, 47). While evidence on the direct connections between extreme heat and the menstrual cycle is limited, hormonal imbalances do affect the menstrual cycle, causing irregularities, atypical bleeding, and intensifying menstrual pain (48, 49). Further, endocrine disorders (like polycystic ovarian syndrome and endometriosis), and menopausal symptoms may be affected by slow onset climate events (like temperature rises) in ways that are understudied (50). Climate related food insecurity affects nutritional intake, which could affect menstruation through anaemia and other nutritional deficiencies (11).

Nascent evidence suggests that chronic exposure to greenhouse gas emissions, a contributor to climate change, may affect menstrual health through pollutants' disruptive effects on the hormone regulating endocrine system (51, 52). Worrying implications for menstrual health include earlier onset of menarche and menopause, atypical changes to the duration of the menstrual cycle (shortened or lengthened beyond the normal range), and menstrual disorders such as polycystic ovarian syndrome - all of which have long term health considerations (53, 54). Early onset menarche, for instance, may increase the risk for poor bone and cardiovascular health, mental health and fertility issues in adulthood (53).

#### 3.1.3 Climate change intensifies inequalities and vulnerabilities, stigma and discrimination

Climate change exacerbates challenges to MHH through its negative impact on gender and social inequalities, and resultant discriminatory social norms and taboos imposed on girls and women, including issues such as displacement, economic hardship and GBV (55, 56). Climate related migration results in girls and women losing access to essential and familiar services for information, menstrual products, WASH and health care facilities, and may intensify mobility restrictions. In Chaing Rai and Chain Mai provinces in Thailand, climate change affected water access, potentially compromising MHH among girls and women (57). Health seeking behaviours for menstrual concerns are poor in non-emergency settings, with menstrual health needs even less likely to be expressed and addressed in challenging contexts. Climate change affects household incomes due to displacement, migration, or through the negative impacts on livelihood activities. With restricted household incomes, menstrual product needs are overlooked and unmet. Girls and women may further de-prioritize their menstrual needs in lieu of safety and other essential family needs (28, 37).

#### 3.1.4 Menstrual products and climate change

All menstrual products have environmental implications throughout the value chain from the sourcing of raw materials, manufacturing, usage, to their end of the life treatment as waste products (58–60). Single-use pads and tampons have higher carbon emissions along this value chain compared to reusable pads and underwear, as well as menstrual cups and discs. The manufacturing process of single use products is energy intensive and requires wood pulp, plastics and other chemicals, and uses fossil fuels for production activities (60). Waste management of used pads and tampons in incinerators and burning chambers is also energy intensive and can be polluting as combustion-based solutions in LMICs are often inefficient (61, 62). Reusable cloth pads are made from cotton and/or synthetic materials, and most menstrual cups are made of medical grade silicone; the production of all these materials have environmental implications (e.g., cotton production is water intensive). The transportation of menstrual products to sales and distribution points within and between countries is another consideration, with single use products carrying a heavier carbon footprint given their popularity and widespread use in relation to reusables (58–60, 63). However, access to affordable and appropriate menstrual products of choice are essential for menstrual health and dignity. Given these considerations, the onus for the carbon footprint of menstrual products, and the responsibility for actions to reduce their carbon footprint should be on manufacturers, regulators and policy makers, and not on users.

### 3.2 Actions to improve menstrual health and hygiene in the context of climate change

There is considerable work done to integrate and prioritize MHH in emergency preparedness and response, such as the Sphere standards for MHH, and a dedicated MHM in emergencies toolkit, both widely used by the sector (25, 64). As climate change poses additional threats to MHH, new or adapted solutions and approaches are needed that build on the existing body of interventions and insights.

We borrow from existing definitions of climate change adaptation and climate-resilient WASH to conceptualize climate-resilient MHH services as those that anticipate, respond to, cope with, recover from, adapt, or transform in response to climate-related events, trends and disturbances, all while striving to achieve and maintain equitable access to MHH services. This means that MHH interventions are climate risk-informed, services are designed to be reliable at all times (during seasonal variability and extreme weather events), and are sufficiently robust to ensure the longer-term sustainability of the services and infrastructure.

#### 3.2.1 Access to menstrual products and materials

The most common MHH intervention during disasters is the provision of menstrual products for hygienic management when regular supplies are inaccessible due to inadequate availability, displacement, or economic hardship. In response to acute climate events, a range of products should be available in shelters, schools and healthcare facilities, and should be included in non-food item distributions. In disaster contexts, dignity and hygiene kits, comprising single-use sanitary pads, and in some contexts reusable cloth or readymade cloth pads, have been stockpiled and distributed to vulnerable regions, where culturally appropriate (25, 37, 65). Kits also contain underwear, towels, bathing soap, washing detergent, and clothes pins to enable the washing and drying of underwear and reusable materials (25, 37, 64, 65).

Composable single-use pads, reusable pads, and menstrual cups may be introduced in communities facing protracted climate events, alongside comprehensive information, usage and disposal support. Girls and women need to be consulted to ensure that appropriate menstrual products are selected for distribution, and usage should be demonstrated for new and unfamiliar products. Direct feedback from users need to be collected to ensure that products are context relevant and safely used in constrained settings (37). Given limited documentation, case studies and research are needed to support the appropriate introduction and sustained hygienic use of reusable products like menstrual cups in climate vulnerable communities.

A climate resilience approach to sustained product access includes assessing, establishing and strengthening menstrual product supply chains for acute and long-acting climate events. Strategies in regions that face high burdens of climate change is crucial, and can include supporting local manufacturers, vendors, and brand owners to procure or manufacture sufficient quantities to meet local needs, developing and maintaining storage and distribution infrastructure (65, 66). Market development in climate vulnerable countries need to focus on a range of affordable quality products that meet expressed needs and are culturally acceptable, and that enable users to cope with or adapt to climate events safely and with dignity (36). The ongoing efforts by the International Standards Organization on harmonizing product standards, both global and national (ISO TC 338) can consider additional aspects related to climate risks and environmental impacts of different types of products. Furthermore, tax reforms for menstrual products should be prioritized for countries that experience high climate risk to make affordable quality products consistently available (36).

#### 3.2.2 Imparting knowledge and skills

Acute climate events require essential information on hygienic menstrual management within constrained contexts. Hygiene and dignity kits handouts contain basic information in textual and/or pictorial form (25). Comprehensive awareness sessions with educational materials can be implemented for communities facing chronic or repeated climate events (e.g., annual cyclones), and for displaced communities living in camps or settlements for extended periods of time. These sessions, coupled with peer support groups, can help communities anticipate and prepare for MHH needs in the short and mid-term. Preparedness actions include keeping sufficient quantities of menstrual products, using menstrual products that are convenient during and after disasters, preparing young girls for menarche and puberty, procuring sufficient medication for pain or menstrual concerns, and supporting caregivers of girls and women with disabilities as has been done in Vanuatu (67). Awareness sessions should be delivered by sensitized and trained facilitators in (temporary) schools or healthcare facilities, identified safe spaces, and at menstrual product distribution points (37). Digital apps or chatbots, such as Oky or Probahini, can be used to reach people with information via smart phones before, during and after climate disasters. Guidance on managing menstrual pain, discomfort, disorders, and menopausal symptoms can be provided in communities facing slow onset climate events or recurring disasters, but few examples exist on how this has been done thus far. Training and educational materials for community health workers and school nurses could be reviewed to include information on anticipated effects of climate change to the menstrual cycle. Any and all information imparted needs to be risk informed and tailored to the specific climate event, trend or disturbance, and the effects it may have on MHH.

#### 3.2.3 Access to climate-resilient and menstrual-friendly facilities and services

Climate-Resilient WASH services anticipate, respond to, cope with, recover from, adapt to or transform based on climate-related events, trends and disturbances, all while striving to achieve and maintain universal and equitable access to safely managed services, even in the face of an unstable and uncertain climate, where possible and appropriate, minimizing emissions, and paying special attention to the most exposed vulnerable groups (31). Specifically, for MHH, continued access to toilets, water and soap for personal hygiene, and access to a safe and private space to wash and change is important. Examples of climate resilient WASH facilities are elevated toilets in flood affected areas or solar-powered water supply systems (68). Menstrual waste disposal solutions are needed, especially in flood and cyclone situations and in temporary settlements (36). As governments, organizations, and communities build climate resilient WASH systems, incorporating a gender lens in WASH standards will support responses to the unique needs of girls and women. The MHM in emergencies toolkit provides guidance on gender responsive WASH facilities, highlighting solutions for washing, laundering and waste disposal in constrained contexts (24, 25).

Additionally, the resilience of institutions that provide menstrual services, such as healthcare facilities and schools, need to be prioritized, or temporary arrangements need to be made until systems are restored. Policies and implementation guidelines need to clearly incorporate MHH in climate resilient education and healthcare systems, and while efforts for this have started, for example in Honduras, Tajikistan, Vietnam and India, their success remains undocumented.

#### 3.2.4 Social support for menstrual health

Social support initiatives to end menstrual stigma and discrimination must be climate risk informed, with special attention to those that are exacerbated during climate events. Girls and women should be consulted to understand their MHH practices and cultural beliefs, and how climate events affect them. Specific attention should be given to marginalized populations such as displaced people, refugees, people with disabilities, and indigenous groups to ensure they have equitable access to MHH resources, education, and support systems. Regular programme and communication approaches that address stigma and provide social support can then be adapted to the specific climate context, and intervention teams can be sensitized and trained accordingly (25, 37). Examples of how social support for MHH has been built in lieu of climate change have not been documented.

#### 3.2.5 Policy interventions

Climate resilient MHH services calls for evidence-based advocacy with policy makers, donors, development sector actors and community leaders. MHH considerations should be included into disaster preparedness and response plans, as well as environmental impact assessments to identify local risks, develop mitigation plans and implement tailored interventions (36). In India, for instance, two climate vulnerable states include attention to menstrual health in their disaster response plans through the provision of menstrual products and gender-responsive WASH facilities (65). Policy considerations should consider the incorporation of MHH in NDCs and national adaptation plans, in conjunction with SRHR, climate resilient WASH and healthcare systems. This can open opportunities to include MHH in climate financing plans and proposals.

## 4 Recommendations and conclusion

To meet MHH needs in a world increasingly affected by climate events, the paper concludes that actions focus on the following broad areas:

• Investments in research, data, evidence, and monitoring of the intersections of MHH, SRHR, WASH and climate change to inform evidence-based interventions, particularly in disaster-prone and climate-vulnerable regions.
• Conceptualization of climate resilient MHH services, and its application in MHH programming and policy actions.
• Cross-sectoral collaboration across stakeholder groups (including girls and women themselves), advocating for the inclusion of MHH in relevant climate and disaster risk reduction policies and plans, while also considering climate resilient MHH as an important component of climate resilient health services, WASH, and other relevant sectoral initiatives.
• Greater discussions on intersectional justice approaches to climate, MHH, SRHR and other justice issues to mitigate the undue disadvantages faced in LMICs.
• Supporting grassroots movements and community organizations to break cultural norms and stigma surrounding MHH and fostering open dialogue on local responses to climate-resilient MHH.
